# Size-Dependent Ability of Liposomes to Accumulate in the Ischemic Myocardium and Protect the Heart

**DOI:** 10.1097/FJC.0000000000000606

**Published:** 2018-06-26

**Authors:** Rinat A. Mukhamadiyarov, Evgeniya A. Senokosova, Sergey S. Krutitsky, Darya V. Voevoda, Inna A. Pyshnaya, Vladimir V. Ivanov, Martin J. Lewis, Igor Khaliulin

**Affiliations:** * Research Institute for Complex Issues of Cardiovascular Diseases, Siberian Branch of the Russian Academy of Medical Sciences (SB RAS), Laboratory of New Biomaterials, Kemerovo, Russia;; † Ecology and Natural Resources, Institute of Biology, Kemerovo State University, Kemerovo, Russia;; ‡ Institute of Chemical Biology and Fundamental Medicine, SB RAS, Laboratory of Bionanotechnology, Novosibirsk, Russia;; § Siberian State Medical University, Laboratory of Biological Models, Tomsk, Russia; and; ¶ Translational Health Sciences, Bristol Medical School, University of Bristol, Bristol, United Kingdom.

**Keywords:** drug delivery, heart, size of liposomes, myocardial accumulation and distribution, cardiac ischemia/reperfusion injury, cardioprotection

## Abstract

Liposomes have the potential to be used for drug delivery. Meanwhile, liposome size may affect their accumulation in the target tissue. We investigated the myocardial accumulation of 2 populations of liposomes (∼70 and 110 nm diameter) during ischemia and their effect on ischemia/reperfusion injury. Isolated rat hearts were subjected to 30 minutes of low-flow ischemia with the liposomes, followed by 30 minutes of liposome-free reperfusion. The liposomes were loaded with the fluorescent dye Nile Red to assess their accumulation in the myocardium. The cardiac functional recovery during reperfusion was evaluated using force–velocity characteristics and coronary flow (CF). Reperfusion injury was evaluated by lactate dehydrogenase release. In addition, CF and contractility were assessed in hearts perfused normally with 70 nm liposomes. There was a 6- and 4-fold greater accumulation of the small liposomes in the myocardium and mitochondria, respectively, compared with the large liposomes. Importantly, even without any incorporated drugs, both populations of liposomes improved functional recovery and reduced lactate dehydrogenase release. However, the smaller liposomes showed significantly higher protective and vasodilatory effects during reperfusion than the larger particles. These liposomes also increased CF and contractility during normal perfusion. We suggest that the protective properties of the liposomes could be related to their membrane-stabilizing effect.

## INTRODUCTION

There is significant scientific interest in the potential of liposomes to provide targeted delivery of drugs to damaged areas of the myocardium after myocardial infarction. This interest stems from the substantial contribution of cardiovascular diseases (CVD) to morbidity and mortality and the consequent need to derive novel treatment strategies.^1^ Contemporaneously, there has been remarkable success in the development of targeted drug delivery using liposomes for the treatment of oncologic pathology.^2^

Accumulated information and technological advances in the development of different types of liposomes, the possibility of incorporation of different biologically active compounds in these particles, and their high transport ability opens new horizons in the creation of novel highly effective pharmacological interventions for the treatment of CVD. A wide variety of biologically active compounds, such as cytokine inhibitors, hormones, enzymes, signaling molecules, and antioxidants among others, have been found to have potential therapeutic value in the management of CVD. However, new pharmacokinetic approaches are necessary for these compounds to produce a beneficial effect. This may include ensuring drug delivery to a therapeutic target, protection of these medicines against biodegradation, and prolonged retention in the circulation.^3–5^ Various nanoparticles are being investigated in the search for a means of targeted drug delivery. Liposomes are probably one of the most promising particles in this respect. Once incorporated in liposomes, drugs can easily penetrate cellular membranes and enter the target cells.^6^ However, the transport capability of liposomes depends on their characteristics, such as the lipid composition of the liposomal membranes, their size, charge, various modifications of the liposomal surface, and inclusion of target molecules. Any modification of the liposomes modulates their properties, their interaction with cells, and efficacy of drug delivery.^7,8^

Importantly, pores can be formed in the capillaries located in the area of inflammation that can be caused by different pathological processes, including myocardial ischemia. These pores may become large enough allowing nanoparticles to penetrate the vascular barrier and accumulate in the affected area. This phenomenon is called the passive targeting of nanoparticles.^9^ The liposomes should be small enough to be able to implement this effect in the area of inflammation. It has been found that, to accumulate in the ischemic area, the nanoparticles should not exceed 200 nm.^8,10^ However, reducing the size of the liposomes may impair their ability to accommodate drugs that need to be delivered to the affected area. Consequently, the choice of the size of nanoparticles as a means of transport is of critical importance. Therefore, in this work, we studied the ability of liposomes of different sizes to accumulate in the myocardium during ischemia to identify the optimal size of these particles for targeting the damaged area of the heart.

A number of agents have been found to be cardioprotective when applied before ischemia. However, it is worth mentioning that the clinical relevance of this kind of intervention is currently restricted to cardiac surgery with cardio-pulmonary bypass as the onset of ischemia is predictable in advance. Clearly, salvation of the myocardium during perfusion in patients in whom acute myocardial ischemia has already taken place has a broader clinical relevance.^11^ Furthermore, the passive targeting may contribute to a greater efficiency of liposome-assisted drug delivery before reperfusion rather than before ischemia. Consequently, in the present work, we assessed the accumulation of liposomes of different sizes in the myocardium and evaluated their effect on ischemia/reperfusion (I/R) injury.

## METHODS

### Preparation and Characterization of Liposomes

Liposomes were prepared by extrusion (extruder supplied by Lipex Biomembranes Inc, Vancouver, BC, Canada) of a suspension of multilamellar vesicles through polycarbonate filters.^12^

The composition of the lipid film included egg lecithin (Avanti Polar Lipids, Inc, Alabaster, AL) and cholesterol (Sigma-Aldrich, St. Louis, MO) in the molar ratio of 7:5. To prepare the liposomes containing a luminescent probe, Nile Red (Molecular Probes, Eugene, OR) dye was additionally injected. The molar ratio of lecithin:cholesterol:Nile Red was 7:5:0.015. Because Nile Red is a lipophilic dye and strongly preserved in a nonpolar region of the liposomal membrane, it can serve as a specific marker of liposomes.^13,14^ The lipophilic components were dissolved in chloroform and placed in the flask of a vacuum evaporator. After solvent removal, the lipid film obtained on the walls of the flask was hydrated with 0.9% NaCl (Sigma-Aldrich) solution by shaking to form multilamellar vesicles. To improve hydration, the vesicles were subjected 10 times to a freeze–thaw cycle.

This suspension was passed through the extruder using polycarbonate filters (Corning/Costar, Corning, NY) with pore sizes of 100 or 30 nm. The resulting preparation was diluted with 0.9% NaCl solution to a lipid concentration of 10 mg/mL.

**FIGURE 1.**
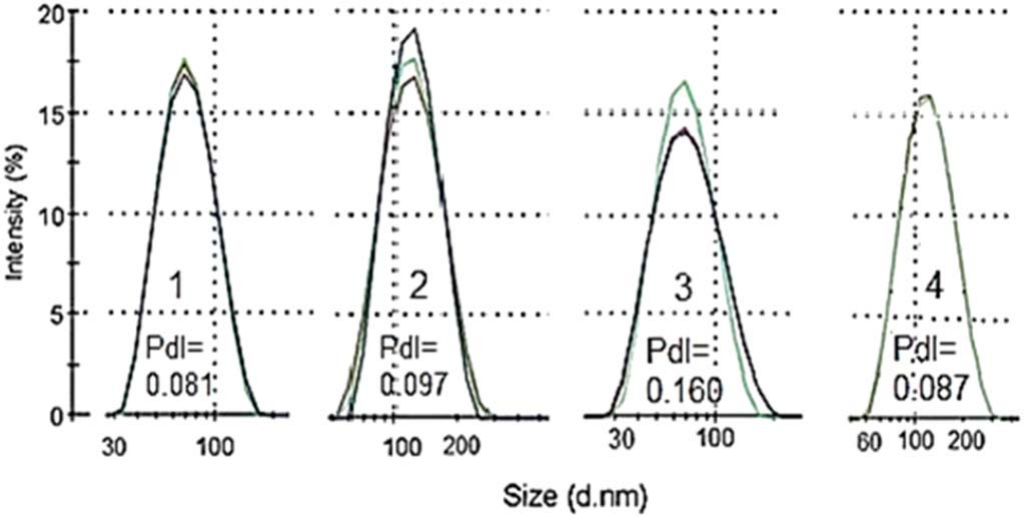
Distribution of the size of liposomes produced using filters with 30 or 100 nm pore diameters (groups L30 and L100). 1, L30; 2, L100; 3, L30+NR; 4, L100+NR. The presented size measurements are the average of 3 samples from each group of liposomes. PdI—polydispersity index (an indication of the width of the overall distribution of sizes).

The size (Z-average diameter, nm) and the polydispersity index (PdI) of the particles in the resulting suspension were determined as an average of 3 measurements for each group of the liposomes using the nanosizer Zetasizer Nano ZS90 (Malvern Instruments Ltd, Malvern, United Kingdom). Pdl is a measure of the distribution of the size of liposomes in a given sample containing the particles. This parameter reflects the width of the overall distribution of particle sizes (Fig. 1).

### Heart Perfusion by Langendorff

All procedures conformed to the Animals (Scientific Procedures) Act, 1986 of the United Kingdom Parliament, Directive 2010/63/EU of the European Parliament and the Guide for the Care and Use of Laboratory Animals published by the US National Institutes of Health (NIH Publication No. 85-23, revised 1996). Ethical approval was granted by the Animal Welfare and Ethics Committee of the Research Institute for Complex Issues of Cardiovascular Diseases, SB RAS, Russia.

Experiments were performed on male Wistar rats weighing 250–300 g. The animals were anaesthetized with intraperitoneal thiopental (50 mg/kg). Sternotomy was then performed; the hearts were excised and then arrested by a brief immersion in ice-cold Krebs–Henseleit buffer (KH) containing (mM) the following: 118 NaCl, 25 NaHCO_3_, 4.8 KCl, 1.2 KH_2_PO_4_, 1.2 MgSO_4_, 11 glucose, and 1.2 CaCl_2_ (chemicals were supplied by Sigma-Aldrich). Following the extraction of the hearts, they were perfused in the Langendorff mode with KH gassed with 95% O_2_ - 5% CO_2_ at 37°C and pH 7.4. The perfusion pressure was maintained at 60 mm Hg.

Force–velocity characteristics, ie, force of contraction (*F*_contr_), speed of contraction (V_contr_) and speed of relaxation (V_relax_), were monitored at a baseline tension of 2 g throughout the experiment using a tension-recording system, custom designed by the Research and Innovation Enterprise “Topaz” (Tomsk, Russia). Lactate dehydrogenase (LDH) activity in the effluent perfusate was measured spectrophotometrically at the wavelength of 340 nm using the method previously described.^15^

### Experimental Protocol

After 15 minutes of equilibration, the force–velocity characteristics of the heart and coronary flow (CF) were measured, and the effluent perfusate was collected for measuring LDH activity. Then, normal perfusion was halted and replaced by a period of 30 minutes of normothermic low-flow ischemia (LFI). LFI was performed by perfusing the heart with nonoxygenated saline solution (0.9% NaCl), with or without the liposomes, using a peristaltic pump at a rate of 0.1 mL/min (less than 1% of the CF measured at the end of the equilibration period). LFI was followed by 30 minutes of reperfusion with KH. After 10 minutes of reperfusion, the CF was measured, and at 20 minutes, force–velocity characteristics were recorded. Effluent perfusate was also collected at 20 minutes of reperfusion for analysis of LDH activity. The effluent perfusate (both with and without liposomes) was discarded at all stages of the experiment.

### Groups of Hearts

The hearts were randomly divided into 3 groups according to the composition of the solution injected during LFI. In the Control group, hearts were injected with nonoxygenated saline solution. Two other groups of hearts were injected with the saline solution containing liposomes of different size, depending on the filters used for their preparation: the group L100 contained hearts that were hypoperfused with the population of liposomes prepared using the filters with 100 nm pore diameter, whereas hearts in the L30 group were treated with the population of liposomes prepared using the filters with 30 nm pore diameter. Nonoxygenated saline solution was chosen to minimize the effects of LFI itself on the pathogenesis of ischemic injury, which includes acidosis, lack of oxygen, and energy substrates.^16^ The characteristics of the 2 groups of liposomes, L30 and L100, are described in the Results section.

An additional series of experiments was performed to assess the effect of liposomes delivered during normal heart perfusion (at the constant perfusion pressure of 60 mm Hg with oxygenated KH) on the force–velocity characteristics and CF. After 15 minutes equilibration period, the heart perfusion was continued with L30 at a concentration of 75 µg/mL for 30 minutes. The force–velocity characteristics and CF were measured at the end of the equilibration period and at 30 minutes of perfusion with the liposomes.

### Evaluation of Liposome Accumulation by the Heart

**FIGURE 2.**
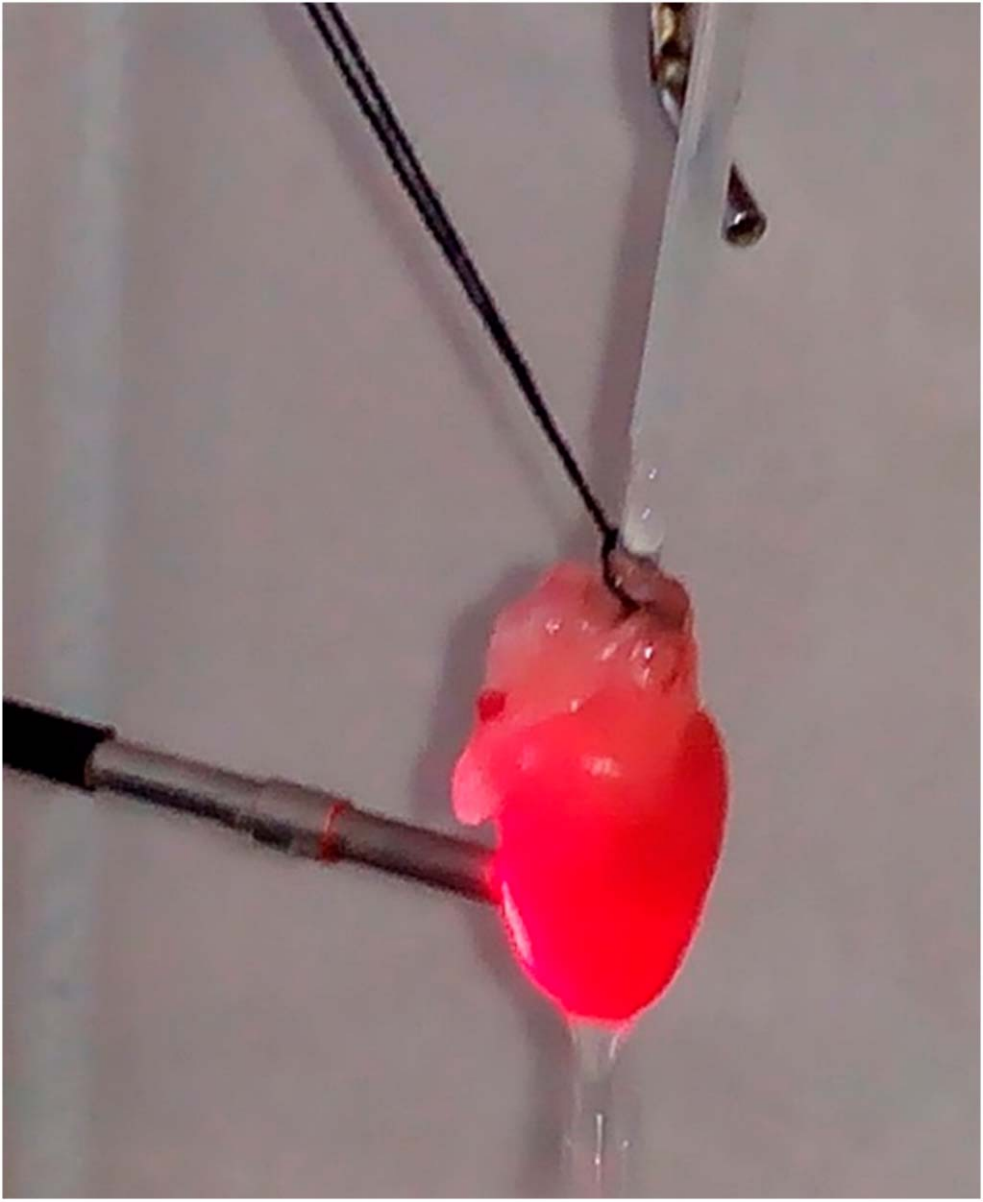
Measurement of surface fluorescence of the heart loaded with the dye Nile Red. A heart loaded with Nile Red was perfused in the Langendorff mode. The fluorescence detection head was placed against the surface of the right ventricle.

After 30 minutes of LFI with saline solution with or without the liposomes containing Nile Red and 10 minutes of reperfusion, the surface fluorescence of the myocardium was measured using the LAKK-M 2 laser diagnostic complex (Research and Production Enterprise SPE “LAZMA,” Moscow, Russia) by placing the fluorescence detection sensor on the surface of the right ventricle (Fig. 2). Then, the heart was removed from the aortic cannula and frozen in liquid nitrogen. The apex of the heart was cut off; cryosections of 10–12 μm were prepared using the Microm HM 525 cryomicrotome (Thermo Scientific, Waltham, MA) and placed on slides coated with l-polylysine (Menzel Glaser, Braunschweig, Germany). The sections were stained with DAPI nuclear stain (Sigma-Aldrich) at a concentration of 10 µg/mL and incubated for 30 minutes at room temperature. The prepared sections were placed into ProLong mountant (Life Technologies, Camarillo, CA) under the coverslip. These preparations were then analyzed using a confocal laser scanning microscope LSM 700 (Carl Zeiss, Aalen, Germany).

For quantification of the content of Nile Red–loaded liposomes in the myocardium, 250 mg of samples were used. The tissue was ground under liquid nitrogen, and Nile Red was extracted by incubation in 2.5 mL of isopropyl alcohol, in the dark, at a temperature of +6°C for 72 hours. After 30 minutes of centrifugation at 3000*g*, the fluorescence of the extracts (Ex/Em 530/570 nm) was measured using a Varian Cary Eclipse spectrofluorometer (Varian Medical Systems, Palo Alto, CA).

To assess the accumulation of liposomes in mitochondria during LFI, evaluation of Nile Red fluorescence was performed in the mitochondrial fraction in the groups of hearts loaded with the fluorescent probe (ie, the groups L100+NR and L30+NR) and compared with the level of fluorescence of the mitochondria isolated from the Control hearts hypoperfused with saline solution only.

### Isolation of Mitochondria and Determination of Liposomal Label

Mitochondria were obtained from 1 g pieces of the heart apex by differential centrifugation (ultracentrifuge Optima MAX-XP; Beckman Coulter, Indianapolis, IN).^17^ The isolated mitochondria were resuspended and extracted in 2.5 mL of isopropyl alcohol (Sigma-Aldrich). Nile Red fluorescence was then measured by a Varian Cary Eclipse spectrofluorometer.

### Statistical Analysis

Data are presented as mean  ±  SEM. Statistical significances of the differences between groups were evaluated by 1-way ANOVA followed by Tukey's multiple comparison post hoc test. The differences between the parameters measured during the equilibration period and reperfusion (or normal perfusion with the liposomes) in the same hearts of each group were assessed using 2-tailed paired Student's *t* test. The statistical analysis was performed using software Statistica, Version 6.0 (TIBCO, Palo Alto, CA). Differences were considered statistically significant where *P*  <  0.05.

## RESULTS

### Characterization of the Liposomes

The characteristics of liposomes of different groups are presented in Table 1. Extrusion of the multilamellar vesicles through filters with a pore diameters of 100 nm (L100) or 30 nm (L30) led to the formation of 2 populations of liposomes with an average diameter of ∼110 and 70 nm, respectively. The maximum and the minimum size of the liposomes of these groups were also different (Table 1). The distribution of sizes in the L100 and L30 groups, with and without the fluorescent dye Nile Red, was assessed by calculating PdI (Fig. 1). This index was similar in groups L30 and L100. Addition of the fluorescent probe Nile Red to the liposomal composition increased PdI in liposomes prepared with the 30 nm filter pore (group L30+NR). However, the average size of the liposomes, both L30 and L100, remained unchanged by Nile Red (Table 1).

**TABLE 1.**
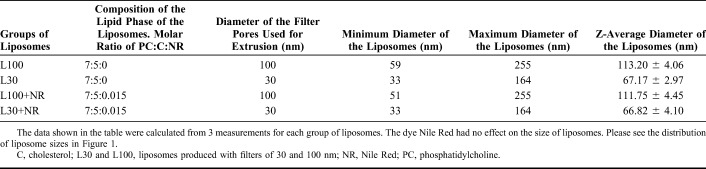
Characteristics of the Groups of Liposomes

### Accumulation of Liposomes in the Myocardium During LFI

**FIGURE 3.**
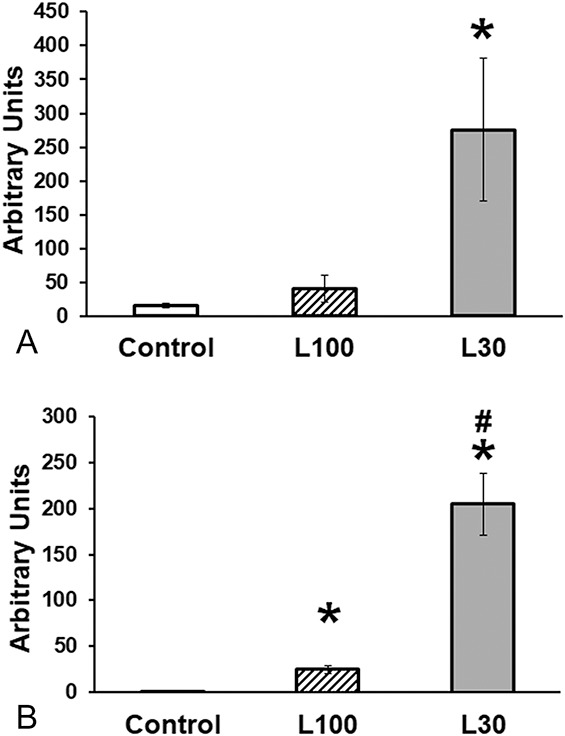
Accumulation of liposomes in the myocardium loaded with the dye Nile Red (NR) during LFI. Liposomes accumulation was measured at 10 minutes of reperfusion. Axis Y—fluorescence intensity of Nile Red. Groups of hearts: Control (n = 6), L30+NR (n = 6), and L100+NR (n = 6). Panel A—surface fluorescence in the hearts after 30 minutes of LFI. Panel B—fluorescence of the hearts homogenized after 30 minutes of LFI. *P < 0.05 versus Control; #*P* < 0.05 versus L100+NR.

Surface fluorescence of the hearts subjected to 30 minutes of normothermic LFI with liposomes of the 2 groups, L30 and L100, loaded with the dye Nile Red showed a remarkably high accumulation of L30 in the myocardium. The fluorescence of hearts treated with L30+NR was 6-fold higher than that in hearts of L100+NR group (Fig. 3A; *P* < 0.05).

**FIGURE 4.**
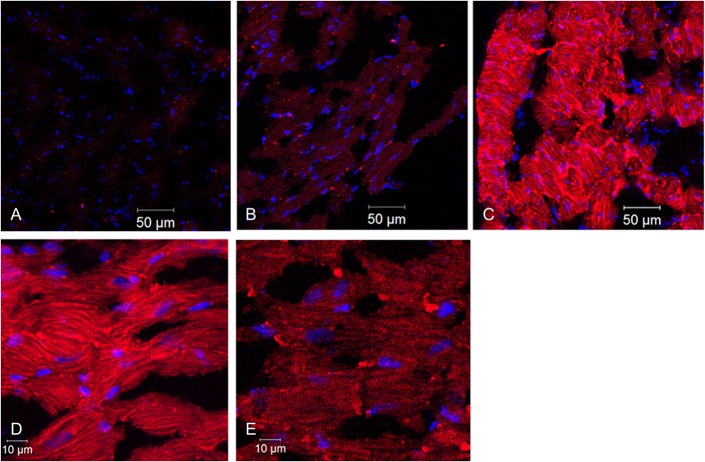
Distribution of the dye Nile Red in the myocardium after LFI with liposomes. Isolated rat hearts were subjected to 30 minutes of LFI with liposomes (L30 and L100 groups, n = 6 in each group) or with saline solution (control group, n = 6), frozen, sectioned, loaded with the dye DAPI, and visualized using a confocal microscope (see methods). Blue fluorescence corresponds to the nuclei dyed with DAPI. Red fluorescence corresponds to liposomes loaded with Nile Red. Panel A, Control; Panels B and E, L100; and Panels C and D, L30.

The level of fluorescence of the isopropanol extracts obtained from the hearts homogenized after LFI showed a similar extent of differences between the groups L30+NR, L100+NR, and Control. Fluorescence of the L100+NR group was significantly higher than that in the Control (*P* < 0.05), whereas fluorescence of the L30+NR group significantly exceeded the fluorescence of both Control and L100+NR groups (Fig. 3B; *P* < 0.05). These differences were also obvious in the confocal images. Thus, the level of fluorescence of the Control hearts, hypoperfused with the saline solution, was very low (Fig. 4A). However, after LFI with L100 and L30 loaded with Nile Red, the level of fluorescence grew considerably (Figs. 4B–E). Particularly notable was the sharp increase in fluorescence in hearts hypoperfused with L30+NR (Figs. 4C, D).

### Localization and Distribution of the Liposomes in the Myocardium

**FIGURE 5.**
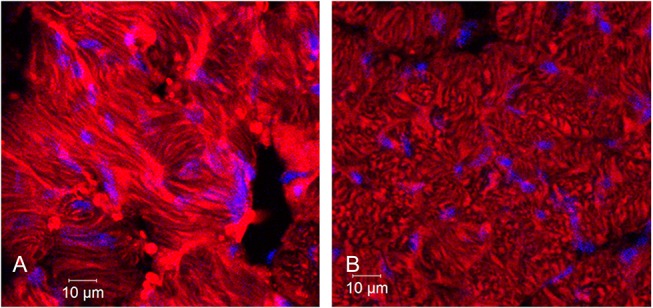
Localization of liposomes after hypoperfusion with L30. Isolated rat hearts were subjected to 30 minutes of LFI with liposomes L30, frozen, sectioned, loaded with the dye DAPI, and visualized using a confocal microscope (see Methods for more details). Blue fluorescence corresponds to the nuclei dyed with DAPI. Red fluorescence corresponds to liposomes loaded with Nile Red. Panel A, Longitudinal section of myofibrils; Panel B, transverse section of myofibrils.

In general, the localization and distribution of the liposomes (assessed by Nile Red fluorescence) in the myocardium was similar to hearts treated with liposomes from each of the 2 groups. Nonhomogeneous fluorescence and the presence of bright granular structures were observed in hearts of the L30+NR group (Figs. 4C, D). In the L100+NR group, a weak level of fluorescence (Figs. 4B, E) made the analysis of localization of Nile Red in these hearts very difficult. In the longitudinal and transverse sections of the hearts hypoperfused with L30, Nile Red fluorescence was located both around and inside of the cardiomyocytes (Fig. 5).

**FIGURE 6.**
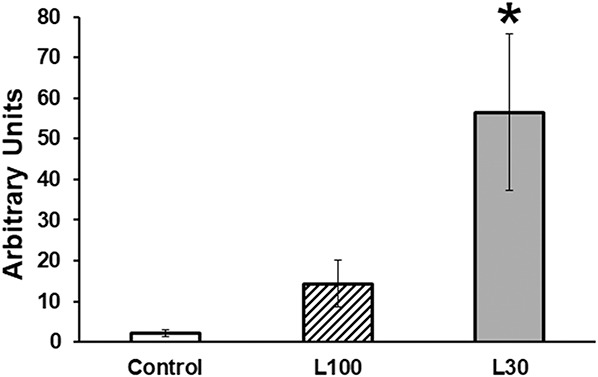
Fluorescence of Nile Red in mitochondrial fraction of the myocardium. Isolated rat hearts were subjected to 30 minutes of LFI with liposomes (L30 and L100 groups) or with saline solution (n = 6 in each group). After LFI, hearts were homogenized, and mitochondria were isolated using differential centrifugation as described in Methods. *P < 0.05 versus Control.

Fluorescence of the mitochondrial fraction was observed in both groups hypoperfused with liposomes. However, in the hearts treated with L30+NR, the level of fluorescence was 4-fold higher than that in hearts hypoperfused with the L100+NR group (Fig. 6).

### Functional Recovery of the Hearts During Reperfusion

**FIGURE 7.**
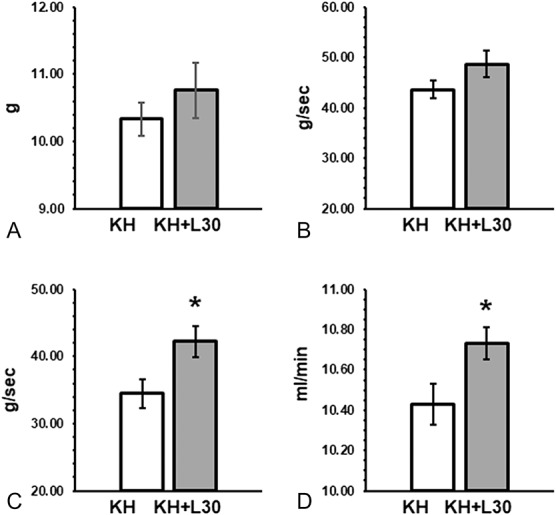
Effects of normal KH perfusion with liposomes (L30) on the force–velocity characteristics and CF. Isolated rat hearts (n = 8) were perfused normally with oxygenated KH at 60 mm Hg. After the 15-minute equilibration, L30 were added to the perfusate at the final concentration of 75 µg/mL. *F*_contr_ (Panel A), V_contr_ (Panel B), V_relax_ (Panel C), and CF (Panel D) were measured at the end of equilibration and at 30 minutes of perfusion with L30. *P < 0.05 versus KH perfusion without liposomes.

Normal perfusion of hearts with oxygenated KH containing L30 (75 µg/mL) resulted in a slight but significant increase in CF that was accompanied by a significantly increased V_relax_ (Fig. 7). *F*_contr_ and V_contr_ also tended to be elevated in these hearts during perfusion with the liposomes.

Analysis of both the recovery of contractile function and of CF also revealed significant differences between the groups of hearts treated with L30 and L100 (Table 2). After LFI with the saline solution (Control group), the force of contraction (*F*_contr_) recovered to 53% of the preischemic value after 30 minutes of reperfusion. In the L100 group, this parameter recovered to 67% (*P* > 0.05 vs. Control), and in the L30 group, *F*_contr_ reached 82% of the preischemic value (*P* < 0.05 vs. Control).

**TABLE 2.**
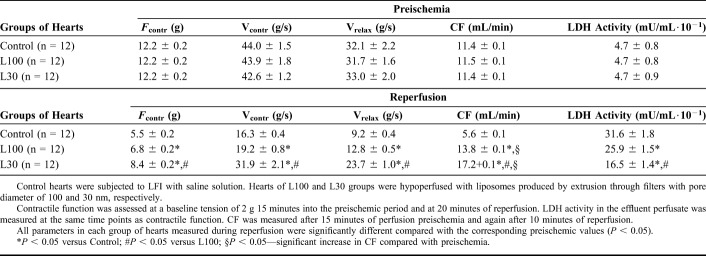
Effects of Low-Flow Ischemia With Liposomes of Different Sizes on the Force–Velocity Characteristics, CF, and LDH Release During Reperfusion

Recovery of the speed of contraction (V_contr_) was also different between the groups. In the Control group, V_contr_ decreased to 37% of the preischemic value, whereas in the L30 group, this index was twice as high as in the Control group. In L30 hearts, V_contr_ was 29% greater than that in the hearts treated with L100 (*P* < 0.05). Similar differences between the groups were observed for V_relax_ (speed of relaxation). In the L30 group, this parameter after reperfusion seemed to be significantly higher compared with both Control and L100 hearts (*P* < 0.05, Table 2).

The differences in CF were even greater between the groups (Table 2). In the hearts of the Control group, CF fell nearly 2-fold after 20 minutes of reperfusion compared with the preischemic value. Meanwhile, in hearts hypoperfused with liposomes, CF seemed to be increased even compared with the preischemic values. In the hearts of the L100 group, this parameter was elevated by 20% (*P* < 0.05), whereas in the L30 group, CF grew by 50% of the preischemic value (*P* < 0.05). This was accompanied by the lowest activity of LDH in the effluent perfusate sampled after 20 minutes of reperfusion, where LDH activity in the L30 group was 2-fold lower than that in the Control (Table 2).

## DISCUSSION

The results of this study confirmed the ability of liposomes to penetrate the capillary barrier in the heart during myocardial LFI and accumulate in the myocardium. Importantly, the degree of accumulation of liposomes depended on their size. In our experiments, L30 (average liposome diameter of ∼70 nm) seemed to be able to accumulate in the ischemic myocardium with a significantly higher efficiency than L100 (average liposome diameter of ∼110 nm).

Liposomes of a variety of sizes have been used in studies investigating the transport of liposomes to the myocardium. In particular, Verma et al^18^ used liposomes of ∼170–190 nm before 30 minutes of normothermic ischemia of isolated rat heart. In this work, the authors demonstrated accumulation of liposomes in the area of infarction. In another study,^19^ liposomes of ∼130 nm containing adenosine were administered in the bloodstream at the end of regional ischemia. This treatment resulted in a significant reduction of the infarct size. Paulis et al^20^ have compared the distribution of micelles (∼15 nm) and liposomes (∼110 nm) loaded with a fluorescent dye. They found that micelles accumulate in the myocardium during the acute stage of infarction but can be washed out quickly from this zone. The rate of penetration of the liposomes to the infarcted area was slower but they remained in the myocardium for longer. Previously, we have shown the ability of liposomes of ∼70 nm loaded with antioxidants to attenuate I/R injury.^12,21,22^ Interestingly, in that study, even the liposomes without antioxidants induced a cardioprotective effect.

Clearly, reduction of liposome size would enhance their effect of passive targeting to the ischemic myocardium. However, this would inevitably diminish their capacity to carry the drugs in either the core or in the membrane of the liposomes. The results of our experiments confirmed that the ability of the population of relatively small liposomes, produced by extrusion through 30 nm filters (the diameter of most of these liposomes was ∼70 nm, as seen from Table 1), to accumulate in the ischemic myocardium is significantly higher compared with the population of larger particles, prepared using 100 nm filters (with an average liposomal diameter of ∼110 nm). This effect will, in our belief, need to be taken into account when the liposomes are used for targeted drug delivery.

In our experiments, liposomes were able to preserve the contractile function of hearts after LFI. This protective effect of liposomes was in accord with previously published data on the antiarrhythmic effect of liposomes. Soloviev et al^23^ have shown that quercetin-filled phosphatidylcholine liposomes are able to prevent peroxynitrite-induced cardiac arrhythmias in the models of isolated rat papillary muscle, Langendorff-perfused rat hearts, and anesthetized rats. Unfortunately, this study lacked a group of “empty” liposomes (without any additional drugs incorporated in the liposomes). However, recently, we have found that “empty” L30, as well as L30 loaded with antioxidants (vitamin E and superoxide dismutase), can effectively prevent ventricular arrhythmias when administered intracoronary before ischemia.^12^ The effect of liposomes on cardiac rhythm is an important area of research that needs further investigation.

In contrast to hearts hypoperfused with the saline solution, liposomes prevented the “no reflow” phenomenon after LFI. The CF is usually regulated by the needs of myocardial contraction such that the perfusate supply is matched to its demand.^24^ Accordingly, CF in Control hearts was significantly reduced during reperfusion. The improved postreperfusion contractility in hearts of the L30 group compared with the Control may partially explain the increased CF in these hearts. However, this parameter was not only preserved but even significantly increased in the liposome-treated hearts compared with the preischemic value. Furthermore, when L30 were perfused in normal conditions in our experiments, they also increased CF resulting in the increased contractile function of the myocardium. These results imply that LFI of hearts with liposomes in our experiments induced a vasodilatory effect that was independent of the myocardial needs during reperfusion. Indeed, we have shown previously that recovery of CF in hearts treated with liposomes can be associated with increased activity of the endothelial nitric oxide synthase, which brought about vasodilation and endothelial protection by reducing lipid peroxidation.^21^

According to the data obtained with the laser diagnostic complex and spectrofluorometric evaluation of fluorescence of the homogenized hearts, intense fluorescence was observed in Nile Red spectrum of the hearts treated with the liposomes loaded with the dye. We therefore used Nile Red as a liposomal label. Our results then indicate that LFI of hearts with liposomes of either size provides successful transport of these particles into the ischemic myocardium. Remarkably, the accumulation of L30+NR in the ischemic myocardium dramatically exceeded the accumulation of L100+NR. Both methods used to evaluate uptake of the liposomes by the tissues showed approximately a 6-fold increase in the efficiency of particle delivery to the myocardium when the diameter of liposomes was ∼70 nm compared with the ones with the average size of 110 nm. Based on the results acquired using confocal microscopy (Figs. 4B, E vs. Figs. 4C, D), the fluorescence of the samples in the Nile Red spectrum in the L30+NR group was significantly higher than that in the L100+NR group at the same settings of the confocal microscope.

Differences in fluorescence between different cardiomyocytes were observed in heart sections from the L30+NR group. Because in cardiomyocytes the mitochondria are localized along the myofibers, the observed fluorescence of Nile Red in this region can be partly provided by the labeled liposomes absorbed by these organelles. To verify this assumption, we isolated mitochondria from the hearts of the Control, L30+NR and L100+NR groups and estimated the content of Nile Red. The results from this experiment proved the presence of the labeled liposomes of both liposome-treated groups in the mitochondria (Fig. 6). However, in the L30+NR group, the fluorescence of Nile Red was 4-fold higher than in the L100+NR group.

The presence of the liposomal label in the mitochondria after LFI with liposomes is of great importance because these organelles play an important role in the mechanism of I/R injury of the myocardium.^16^ Delivery of mitochondria-targeted biologically active substances (eg, antioxidants) may contribute significantly to the preservation of mitochondria and reduce myocardial injury in general.^25^

Thus, these results prove the possibility of successful delivery of liposomes to the ischemic myocardium and that the size of these liposomes is critical for targeted delivery to this area of the myocardium. Indeed, to achieve the protective effect, the liposomes must overcome the vascular barrier formed by endotheliocytes and basal membrane and penetrate the targeted area.^26^ Therefore, during systemic or local administration of the liposomes, the effect of passive targeting is of paramount importance.

Liposomes could be administered by intracoronary infusion during cardiac surgery with cardioplegic arrest and cardiopulmonary bypass or during heart preservation before transplantation. In these cases, heart would be the only organ where the liposomes are delivered, and these nanoparticles would be accumulated in the affected areas of the myocardium because of the effect of passive targeting. During systemic administration of liposomes as drug carriers, they may be degraded by macrophages, which would shorten their circulation in the body. However, this problem can be solved by supplementing the composition of the liposomal membrane with polyethylene glycol.^27^

When the optimal size of the liposomes is established, targeting of these particles can be further adjusted by modifying their other properties. Thus, by changing the characteristics of the liposomes, it is possible to provide not only their targeted delivery to a certain type of cells, but also target them to certain cellular organelles within those cells, including the mitochondria. This can be achieved by inclusion of antibodies, ligands of specific membrane receptors of the target tissues,^28^ or other molecules able to target these nanocarriers to the mitochondria.^29,30^ Interestingly, our data revealed that even the ordinary nonactively-targeted liposomes used in our experiments are delivered into the mitochondria. It would be important to study the effects of liposomes on mitochondria because these organelles play a crucial role not only in energy metabolism, but also in regulation of the mechanisms of cell death and survival.^16^

Importantly, in our experiments, liposomes served not only as transport containers but also as independent therapeutic agents. The mechanism of the cardioprotective effect of liposomes has yet to be discovered. However, some previously obtained data may shed light on the possible mechanism of this effect. Our liposomes were composed of lecithin, a mixture of glycerophospholipids (PLs), and cholesterol, an essential structural component of biological membranes. Küllenberg et al^31^ have pointed out that PLs have a positive impact in several diseases, eg, immune or cancer cells, apparently without severe side effects. The authors noted that these properties of liposomes can partially be explained by the fact that PLs are highly effective in delivering their fatty acid residues (including cholesterol) for incorporation into the membranes of cells involved in different pathologies. It can be suggested that the liposomal membrane can act as a donor of lipids or even fragments of “membrane patches” for damaged organelles^32^ and optimize the lipid balance, ameliorating the membrane damage caused by LFI in our experiments. This membrane-stabilizing effect of liposomes could also contribute to the prevention of cardiac arrhythmias mentioned above.^12,23^ Because the uptake of L30 by the ischemic myocardium was notably higher than that of L100, and this was accompanied by a stronger cardioprotective effect in L30 group, we assume that the beneficial effect of liposomes in our experiments was dose dependent.

In most of the studies on the targeted delivery of liposomes to the ischemic myocardium, liposomes equal to or greater than 100 nm were used. This was probably associated with the convenience of preparation of this kind of liposomes by extrusion through polycarbonate membranes. Moreover, liposomes of this size have a sufficiently large internal volume for the delivery of biologically active substances. However, the results presented in our study show that reducing the particle size to ∼70 nm (group L30) improves the efficiency of their delivery by 6-fold compared with the liposomes of ∼110 nm (group L100).

Thus, liposomes represent a promising means of targeted drug delivery according to the experimental data, including the results of the present study performed on a rat heart model. However, data obtained in the experiments on small rodents cannot be extrapolated directly to clinical practice. To advance the translation of these findings, experiments on isolated human tissues or the large animal models can be used. For example, porcine heart has anatomy and physiology very similar to the human heart and ideal for the purpose of translation.^33,34^

## CONCLUSIONS

Taken together, our experiments reveal that the liposomes composed of egg lecithin and cholesterol possess pharmacokinetic and pharmacodynamic properties, which make them a very promising means for the treatment of CVD.

These liposomes can accumulate in the ischemic myocardium and induce their own protective effect against ischemia/reperfusion injury. This protective effect is manifested in better preservation of myocardial contractile function, prevention of the no-reflow phenomenon, and reduction of LDH release during reperfusion, implying that liposomes possess a membrane-stabilizing effect. Interestingly, the liposomes also induce a pronounced vasodilatory effect in the myocardium.

Importantly, the cardioprotective and vasodilatory effects of the liposomes depend on the size of these particles: the population of liposomes with an average diameter of ∼70 nm demonstrates a more marked protection than the population of liposomes with an average diameter of ∼110 nm. These differences of the effects of liposomes of different size are dose dependent because the smaller particles may penetrate the capillaries more easily and accumulate in the ischemic myocardium.

A fraction of the liposomes delivered to cardiomyocytes are localized in the mitochondria, which may further potentiate the protective activity. The accumulation of these particles in the mitochondria is also increased when the size of liposomes is reduced. Therefore, we believe that liposomes with an approximate diameter of 70 nm can act as an effective transport platform for delivery of drugs to the ischemic myocardium and may be used to create novel highly effective pharmacological interventions.
